# Reinforcing Effects of Calcium Silicate-based Cement and Dual Cure Composite Resin in Simulated Immature Teeth with an Open Apex: An *in vitro* Study

**DOI:** 10.5005/jp-journals-10005-1464

**Published:** 2017-02-27

**Authors:** Murtuza S Zhabuawala, Roopa R Nadig, Veena S Pai, Yashwanth Gowda, Ranjini M Aswathanarayana

**Affiliations:** 1 Postgraduate Student, Department of Conservative Dentistry and Endodontics Dayananda Sagar College of Dental Sciences, Bengaluru Karnataka, India; 2 Professor and Head, Department of Conservative Dentistry and Endodontics Dayananda Sagar College of Dental Sciences, Bengaluru Karnataka, India; 3 Reader, Department of Conservative Dentistry and Endodontics Dayananda Sagar College of Dental Sciences, Bengaluru Karnataka, India; 4 Senior Lecturer, Department of Conservative Dentistry and Endodontics Dayananda Sagar College of Dental Sciences, Bengaluru Karnataka, India; 5 Reader, Department of Conservative Dentistry and Endodontics Dayananda Sagar College of Dental Sciences, Bengaluru Karnataka, India

**Keywords:** Biodentine, Ca/P ratio, Dental trauma, Fracture resistance, Immature teeth, Paracore.

## Abstract

**Aim:**

To evaluate the fracture resistance of simulated immature teeth with an apical plug of biodentine followed by composite resin vs total obturation with biodentine tested immediately and after 3 months of aging and also to find out the chemical composition of dentin in contact with these materials.

**Materials and methods:**

Extracted human maxillary central incisors with simulated immature apex with radicular dentin thickness (RDT) of 1 to 1.5 mm selected and divided into three groups of 20 each. Group I (control)—4 mm biodentine apically and thermoplasticized gutta-percha. Group II—4 mm biodentine apically and composite resin. Group III—complete obturation with biodentine. About 10 samples from each group were tested immediately and remaining 10 stored in phosphate buffered solution (PBS) and tested after 3 months for fracture resistance and chemical analysis of dentin.

**Results:**

No significant difference in fracture resistance between the groups was observed when tested immediately. After 3 months of aging, only biodentine group showed a significant reduction in fracture resistance with increased Ca/P ratio of root dentine.

**Conclusion:**

Biodentine group has shown drastic reduction in fracture resistance after 3 months of aging, and hence, cannot be recommended as a reinforcement material in immature teeth with thin dentin walls.

**How to cite this article:** Zhabuawala MS, Nadig RR, Pai VS, Gowda Y, Aswathanarayana RM. Reinforcing Effects of Calcium Silicate-based Cement and Dual Cure Composite Resin in Simulated Immature Teeth with an Open Apex: An *in vitro* Study. Int J Clin Pediatr Dent 2017;10(4):351-357.

## INTRODUCTION

Dental impact injuries are commonly associated with children between 8- and 12-years-old developing maxillary anterior teeth.^1-3^ These injuries often lead to pulpal necrosis leading to incomplete root development with an open apex, thin dentinal walls, and wide funnel-shaped canal.^4^ Management of such cases is a significant challenge both endodontically and restoratively due to lack of adequate apical constriction and presence of thin dentinal walls.^5^ The traditional approach to the treatment of non-vital teeth with incompletely developed roots has been apexification using calcium hydroxide.^4,6^Andreasen et al^7^ showed that immature roots which had calcium hydroxide placed within the root canals of immature teeth over 1 year had 50% reduction in strength because of proteolytic reaction.

Revascularization has shown great potential for clinical success, although there is no established success rate for regenerative procedures. Garcia-Godoy and Murray^8^ stated that regenerative endodontic treatment is not recommended for patients younger than 7 years or older than 16 years.

Apical placement of mineral trioxide aggregate (MTA) and completion of a procedure in single appointment have been advocated as an promising alternative treatment option with high clinical success rate.^9-11^ Mineral trioxide aggregate has a good sealing ability, high degree of biocompatibility, low cytotoxicity, antimicrobial properties, ability to set in the presence of moisture, shorter treatment time, and induction of hard tissue deposition periradicularly.^12^ However, White et al^13^ reported that tooth strength is weakened after exposure to MTA for more than 5 weeks.

Recently, a new calcium silicate-based cement bio-dentine has been introduced which contains tricalcium silicate, calcium carbonate, and zirconium oxide and water-based liquid that has calcium chlorite and water reducing agent. It sets in 12 minutes and is recommended for use as dentine substitute. Similar to MTA, biodentine is biocompatible and when in contact with vital tissues it is said to be biologically active. The compressive strength and elasticity modulus are comparable with dentine and handling characteristics of biodentine is superior to MTA.^14^ Inspite of their favorable characteristics as an apical plug, the fragility of roots due to thin dentinal walls can compromise tooth strength, hence, reinforcement of radicular dentin is of utmost importance in such cases.^15^

Structural strengthening of non-vital immature teeth has received a great deal of attention and different materials and techniques have been examined.^16-19^ Composite resin has the ability to bond to root dentine walls, increasing the strength of the roots. However, it has certain disadvantages like polymerization shrinkage stresses, difficulty in complete curing, and increased c-factor in the root canal system.^20^ An alternative is the use of fiber post with an elastic modulus similar to dentine shown to have “Monoblock” effect.^21,22^ A mismatch between the diameter of post space and the fiber post is a problem, particularly in immature teeth with large root canals.^23^

Further, placing an apical plug of MTA or biodentine followed by placement of composite resin for radicular reinforcement has certain practical difficulties, such as many interfaces between the materials used, multiple treatment visits, removal of extra MTA/biodentine that has adhered to the root dentine may interfere with bonding and an attempt to remove the adhered material might result in unnecessary removal of already weakened radicular dentin. To overcome the above practical difficulties, there have been attempts to fill the entire canal with these materials. A study conducted to evaluate the fracture resistance of simulated immature teeth with biodentine apical plug and backfilling with various materials concluded that backfilling with guttapercha, fiber post, or biodentine does increase the force required to fracture immature teeth.^24^ However, in all of these above studies, testing was done immediately that too with canal wall thickness of >2.5 mm. Stuart et al^25^ suggested that the canal wall reinforcement of teeth with canal wall thickness of 2 mm or more may not be necessary. But most of the studies available in the literature has been done with the canal wall thickness of 2 mm or more. Therefore, evaluation of fracture resistance of rehabilitated teeth with canal wall thickness less than 1.5 mm is crucial and more relevant. Reinforcement of tooth root depends not only on the inherent strength of the obturating material but also on the quality of bond of the material with the root canal dentin and structural alteration that can happen in the material as well as root dentin over a period of time. Hence, this study is undertaken to evaluate the reinforcing effects of dual cure composite resin and biodentine on immature root dentin and also to find out if these materials can bring about any alteration in root canal dentin that can influence the tooth strength with time.

## MATERIALS AND METHODS

**Figs 1A to D:**
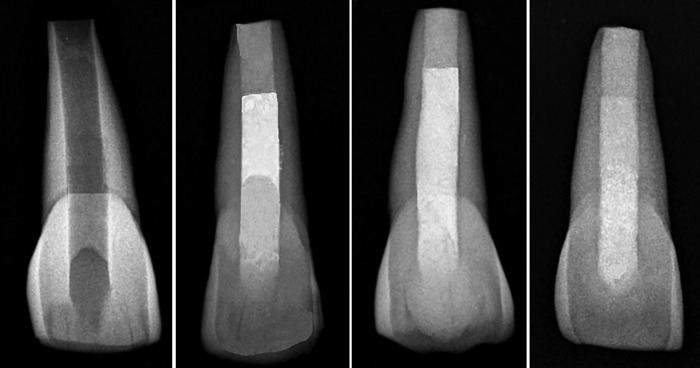
(A) Simulation; (B) gutta-percha; (C) paracore; and (D) biodentine

Sixty extracted human maxillary central incisors measuring 5 mm faciolingually and 6 mm mesiodistally were selected for the study, disinfected with 5.2% sodium hypochlorite for 2 hours, and stored in distilled water until the beginning of the experiment. Preoperative radiographs were taken to rule out any aberrations in the anatomy. The roots of teeth were standardized to a length of 10 mm as measured from the apex to the facial cementoenamel junction (CEJ) by cutting the root tip to simulate incomplete root formation. Endodontic access cavities were made using round bur and Endo Z bur in a high speed hand piece and root canals were prepared using the Protaper rotary instruments. To achieve a simulation of teeth with immature apices, Peeso reamers between #1 and #6 were introduced into the root canal until #6 pass freely out of the apex. After instrumenting with Peeso reamers, the radicular dentin thickness was 2.5 mm. Further 703 carbide bur was used to obtain the remaining dentin thickness around 1.5 mm (Fig. 1). The samples were then subjected to cone beam computed tomography (CBCT) analysis to confirm the dentin thickness. The root canals were irrigated using 3 mL of 3% sodium hypochlorite and final flush with 5 mL of 17% EDTA was carried out to remove smear layer. Biodentine mixed according to manufacturer’s instruction was placed with a carrier and adapted using hand plugger in the 4 mm apical portion of the canal. The teeth were radiographed to verify correct position of the Biodentine. Samples were wrapped in wet gauze, placed in an incubator, and allowed to set for 12 minutes at 37°C with 100% humidity. Specimens were randomly divided into three groups with 20 specimens in each group.

*Group I (n = 20):* AH Plus sealer was applied to the canal walls, backfilled with gutta-percha using Obtura II. Excess sealer was removed from the chamber with dry cotton pellet.

*Group II (n = 20):* Dual-cured composite resin para-core was inserted into the canal and the excess resin was removed and light activation was performed for 40 seconds.

*Group III (n = 20):* Biodentine was mixed and placed to a level just below the facial CEJ.

In all the groups after backfilling procedures, the access openings 2 mm below CEJ were filled with nano-composite (Filtek Z35 0 XT; 3M ESPE, USA). Immediately after filling, 10 samples from each group were randomly divided into two subgroups. In subgroup A: specimens were stored for 1 week in PBS. In subgroup B: specimens were stored in PBS for 3 months until fracture resistance testing. Figure 1 shows radiographs of representative samples of the groups.

### Fracture Testing

To simulate periodontal membrane, the root surfaces were coated with 0.2 to 0.3 mm thick layer of polyvinyl-siloxane impression material and then embedded in the acrylic resin leaving the gap of 2 mm between the top of acrylic and to the CEJ. A custom-made jig was used to fix the cylinders at an angle of 45° and a compressive load was applied from the palatal surface at crosshead speed of 1 mm/minute until fracture using universal testing machine. The maximum force required to fracture each specimens was recorded in Newtons. The data were statistically analyzed using one-way analysis of variance. The level of significance was set at p = 0.05.

### Energy Dispersive X-ray Analysis

After testing fracture resistance, all the specimens were subjected to energy dispersive x-ray analysis to find out the chemical composition of the dentin. The root surfaces were split longitudinally with diamond disk and surfaces were etched with 37% phosphoric acid for 5 second and rinsed thoroughly with water and subjected to elemental analysis under high vacuum at 1,500x magnification using energy dispersive system, which is attached to an environmental scanning electron microscope. The recorded values represented the average mineral weight percentage.

## RESULTS

**Graph 1:**
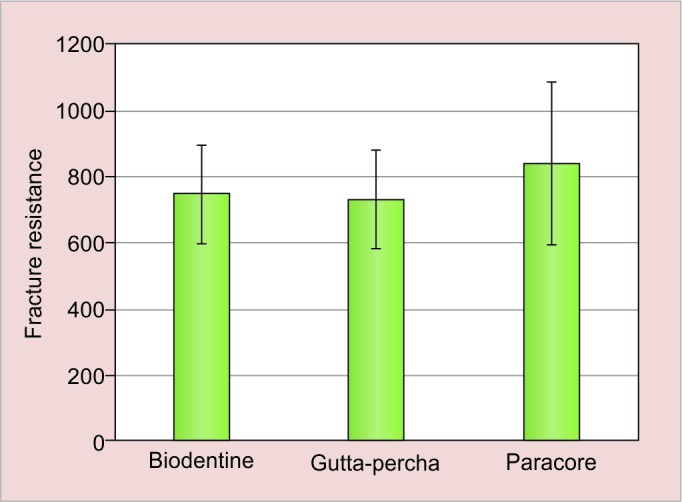
Comparison of fracture resistance between three groups immediately

**Graph 2:**
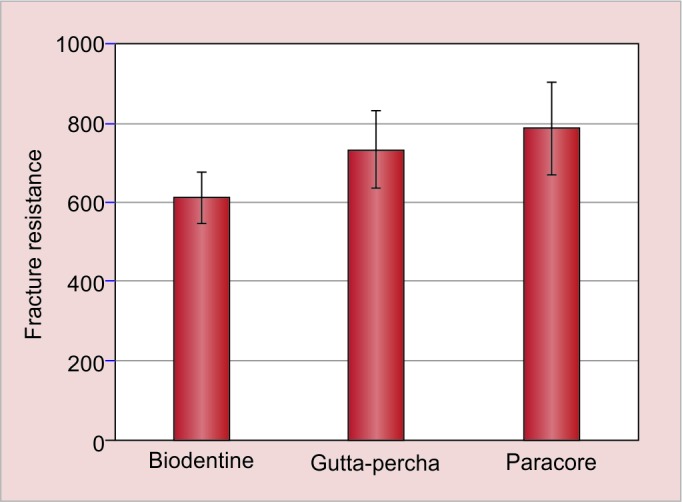
Comparison of fracture resistance between three groups after 3 months

The mean values and their respective standard deviations of the force required to fracture the roots are presented in (Table 1 and Graph 1) and (Table 2 and Graph 2). In immediate tested samples, higher mean fracture resistance was recorded in paracore group followed by Biodentine and gutta-percha group respectively. There was no statistical significant difference in mean fracture resistance between the groups. In delayed tested samples, higher mean fracture resistance was recorded in paracore group followed by gutta-percha and Biodentine group respectively. The difference in mean fracture resistance was found to be significantly different between the groups (p < 0.01). On comparing immediate and delayed testing of Biodentine samples, the difference between them was found to be statistically significant (p < 0.001) where there was no significant difference in other two groups.

**Table 1:** **Table 1:** Mean fracture values for all groups measured in Newtons along with their standard deviations (immediate)

*Groups*		*n*		*Mean forces (n) standard deviation*	
Backfilling with gutta-percha		10		750.450 ± 148.653	
Backfilling with fiber post		10		730.370 ± 147.710	
Backfilling with biodentine		10		841.540 ± 247.782	

No significant differences (p > 0.05)

**Table 2:** **Table 2:** Mean fracture values for all groups measured in Newtons along with their standard deviations (delayed)

*Groups*		*n*		*Mean force (n) standard deviation*	
Backfilling with gutta-percha		10		734.420 ± 97.896	
Backfilling with paracore		10		787.680 ± 116.578	
Backfilling with biodentine		10		612.160 ± 63.799	

No significant differences (p > 0.05)

**Graph 3:**
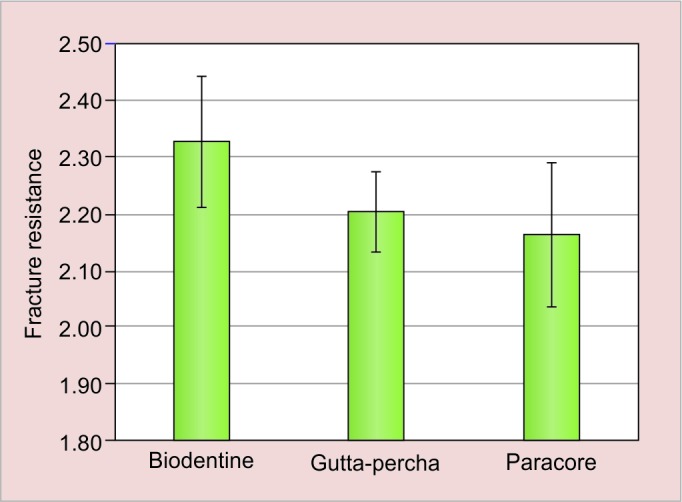
Comparison of Ca/P ratio of dentin between three groups immediately

**Graph 4:**
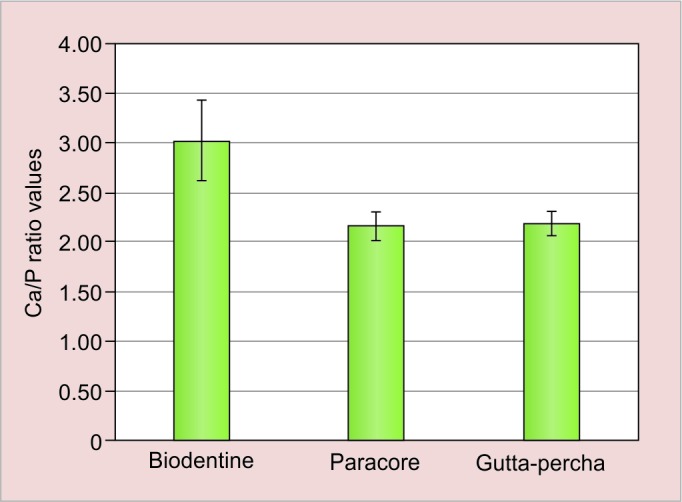
Comparison of Ca/P ratio of dentin between three groups after 3 months

The mean values and their respective standard deviations of the Ca/P ratio of dentin are presented in (Table 3 and Graph 3) and (Table 4 and Graph 4). In immediate tested samples higher mean Ca/P ratio was recorded in biodentine group followed by paracore and gutta-percha group respectively. The difference in mean Ca/P ratio among the groups was found to be statistically significant (p < 0.01). In delayed tested samples higher mean Ca/P ratio was recorded in biodentine group followed by gutta-percha and paracore group respectively. The difference in mean Ca/P ratio among the groups was found to be statistically significant (p < 0.001). On comparing immediate and delayed testing of biodentine samples, higher mean Ca/P ratio was recorded in the delayed specimens compared with immediate specimens and the difference between them was found to be statistically significant (p < 0.001) where there was no significant difference in other two groups.

**Table 3:** **Table 3:** Mean Ca/P ratio for all groups measured along with their standard deviations

								*95% CI for mean*							
*Immediate*		*Mean*		*Standard deviation*		*SE of mean*		*Lower bound*		*Upper bound*		*F-value*		*p-value*	
Biodentine		2.33		0.12		0.04		2.25		2.41		6.216		0.006*	
Paracore		2.21		0.07		0.02		2.15		2.26					
Gutta-percha		2.17		0.13		0.04		2.07		2.26					

*Significant difference

**Table 4:** **Table 4:** Comparison of Ca/P ratio at delayed time interval among the three groups (ANOVA followed by Bonferroni multiple comparisons test)

								*95% CI for mean*							
*Delayed*		*Mean*		*Standard deviation*		*SE of mean*		*Lower bound*		*Upper bound*		*F-value*		*p-value*	
Biodentine		3.02		0.41		0.13		2.73		3.31		35.331		< 0.001*	
Paracore		2.16		0.14		0.05		2.06		2.27					
Gutta-percha		2.18		0.12		0.04		2.09		2.27					

*Significant difference

## DISCUSSION

Reinforcement of endodontically treated immature teeth assumes great importance as these teeth are more susceptible to external and masticatory forces. The selected material for reinforcement must be applied easily in clinical practice, bond successfully to dentine, and serve as a good barrier against microleakage.^25^ Reinforcing capability of biodentine was evaluated in this study because in addition to being bioactive and biocompatible material, it has improved handling characteristics and less time consuming than MTA. Han and Okiji^26^ suggested that BD may have a remarkable biomineralization capability than MTA.

In most of the studies, found in the literature, to simulate immature teeth, the canals were instrumented with Peeso reamers (1-6) until a size 6 Peeso can be passed 1 mm beyond the apex to simulate open apex, this would only result in dentin thickness of 2 to 2.5 mm. Investigations have revealed that the reinforcement of a canal with a diameter less than 1.5 mm is not necessary while wall thickness of 2.63 mm is insufficient to weaken tooth struc-ture.^25^ Therefore, preparation of the canal was further enlarged with 702 carbide burs to approximate Cvek’s stage 3 root development. Cvek’s stage 3 root development was selected for the present model; because at this stage, the root-to-canal ratio in a mesiodistal dimension at the CEJ is nearly 1:1.^15,27^ In the present study, after the simulation of all the samples, CBCT was used to find out the accurate thickness of the remaining radicular dentin.

While testing for fracture resistance, the load was applied at an angle of 45° to the long axis of the tooth to simulate the average angle of contact between maxillary and mandibular incisors in a class I occlusion.^24,25^ On evaluating the results, in immediate tested specimens, teeth restored with paracore group showed the highest fracture resistance followed by biodentine and then gutta-percha. However, there was no statistical significant difference between the groups tested in the study. A preliminary study by Yasir^28^ evaluated the fracture resistance between biodentine and gutta-percha in immature teeth and reported that there was no statistical significant difference between groups. A similar study by Topc.uoglu et al^24^ evaluated the fracture resistance between bioden-tine, gutta-percha, and fiber post group in immature teeth and suggested that there is no significant difference between biodentine and gutta-percha group. In the present study, access composite is extended 2 mm apical to CEJ to provide an additional root strength necessary to minimize cervical root fracture. Seto et al^29^ reported that composite resin to a depth of 3 mm would significantly strengthen the roots in immature teeth. So this could be one possible reason, why there was no statistical significant difference in the fracture strength between the control group and the experimental groups in the current study.

After 3 months of storage, teeth restored with para-core have shown the highest fracture resistance followed by gutta-percha and biodentine showed the least fracture resistance. The difference between immediate and delayed paracore groups, there was a reduction in fracture resistance although it was not statistical significant. Tay et al^30^ reported that the degradation patterns of resin-dentin interface inside the root canal created by self-etching adhesive systems due to water sorption and induced collagenolytic activity adversely affects the longevity of adhesively bonded post. Tay and Pashley^31^ reported that the self-etching systems due to their hydro-philic characteristics tend to present potential degradation in the course of time, as water plays a key role in polymer degradation and concluded that the tested etch-and-rinse adhesive systems had a better performance in terms of bond durability over time than the self-etching adhesive systems and thus monoblock effect is questionable over a period of time. However, our study was of 3 months duration and the longevity of the bond with extended time periods needs further evaluation. The difference between immediate and delayed biodentine group, there is a drastic reduction in the tooth strength after 3 months of storage. Bowes^32^ and Leiendecker et al^33^ reported that highly alkaline calcium hydroxide induces a caustic degradation effect on exposed collagen and this is mediated by the breakdown of intermolecular bonds in collagen fibrils, increasing their water absorption leading to swelling. A recent study by Sawyer et al^34^ reported that dentin flexural strength for dentin exposed to biodentine and MTA decreased significantly after 2 and 3 months. Atmeh et al^35^ examined the dentin cement interfacial interaction with biodentine and reported that in confocal images a layer of mineral infiltration zone is associated with an altered intertubular microstructure leading to change in optical properties of interfacial dentin. SEM micrographs showed the same band of structurally altered dentin immediately beneath the biodentine which is due to high alkalinity of hydrated biodentine, which has induced a caustic denaturing and permeability of organic collagen component of interfacial dentin. Therefore, it is possible that the above reaction might have lead to decreased tooth strength of the biodentine samples after 3 months. Another reason for the decreased tooth strength with biodentine after 3 months of aging could be the solubility of biodentine. Singh et al^36^ have reported that the solubility of biodentine after 30 days and 60 days immersion period was significantly higher as compared with MTA. Vivan et al^37^ theorized that a material releasing calcium ions to exert biologic effect to some extent solubilize and dissociate from fully hardened cement thus resulting in disintegration. In the present study, radiographic examination of the samples observed showed reduction in the radioopacity of biodentine after 3 months of aging which could suggest the disintegration of this material over a period of time. Hence, further evaluation is required for biodentine to be used in apexi-fication procedure. In the present study, radicular dentin of biodentine group has shown a significant increase in the Ca/P ratios from the mean value of 2.33 to 3.02, thus connoting a gain in the mineral content. Thus, these results are in accordance with the previous mentioned study by Han and Okiji^26^ who stated that both biodentine and MTA caused the uptake of Ca and Si in the adjacent root canal dentine in the presence of PBS who reported that Ca and Si uptake in dentin areas. The dentin element uptake was more prominent for biodentine than MTA. Therefore, in the present study, solubility of biodentine could be due to higher uptake of ions by the root canal dentin, which could have altered the physical properties of biodentine and therefore, strength of biodentine itself is reduced over a period of time. It is interesting to note that Hatibovic-Kofman et al^38^ compared the influence of MTA and CH on fracture resistance in sheep teeth and stated that over 1 year period, MTA and CH have showed a 2% and 26% decrease in fracture resistance. Authors reported that after initial decrease in the fracture resistance of MTA treated teeth, the process is reversed, and fracture resistance increases for a period between 2 months and 1 year. It can be explained by the fact that MTA induces the expression of TIMP-2 in dentin matrix and suppress the degenerative activities of MMP-2 and -14. Whether similar phenomenon can be observed in biodentine needs further evaluation.

## CONCLUSION

There was no statistically significant difference in the fracture resistance of an immature tooth with an apical plug of biodentine followed by obturation with gutta-percha, paracore, or biodentine. After 3 months of aging, biodentine group has shown a drastic reduction in the fracture resistance whereas there is no significant reduction in gutta-percha and paracore groups. EDX analysis shows increased Ca/P ratio of root canal dentin of bio-dentine group compared to gutta-percha and paracore after 3 months of aging, whether this uptake of minerals is responsible for decreased tooth strength needs further evaluation. Therefore, biodentine cannot be recommended as a reinforcement material in immature teeth with thin dentinal walls. Further *in vitro* and *in vivo* investigations are necessary to validate the above findings.

## CLINICAL SIGNIFICANCE

- Dental impact injuries involve children at school age between 8 and 12 years can lead to pulpal necrosis with incomplete root formation.
- Management of such cases is a significant challenge endodontically as well as restoratively.
- Structural strengthening of immature teeth has received a great deal of attention and therefore, different materials and techniques have been examined.
